# Supplementary material for: Quantifying microplastic ingestion, degradation and excretion in insects using fluorescent plastics
Source: Conserv Physiol. 2023 Aug 15;11(1):coad052. doi: 10.1093/conphys/coad052 (PMC10425969; doi:10.1093/conphys/coad052)
Supplement: Web_Material_coad052 [file web_material_coad052.zip › Quantifying_microplastic_ingestion_supplemental.pdf]

Table 1S: Counts obtained through automated ImageJ analysis and those derived from manual analysis by of three independent researchers for 15 representative images. ImageJ was set to a threshold of 5 px.

Image	ImageJ count	Researcher 1 counts	Researcher 2 counts	Researcher 3 counts
82 m	10	11	10	11
85 f	27	51	58	56
118 h	8	47	52	55
155 m	6	9	6	9
176 f	79	127	94	183
189 m	60	165	153	228
238 f	37	38	39	47
263 m	22	33	33	50
291 f	124	302	246	287
319 h	57	192	161	260
326 h	9	46	37	45
363 m	63	268	181	292
368 m	19	60	51	76
414 h	12	86	63	144
452 f	3	4	4	6
326 h	9	46	37	45

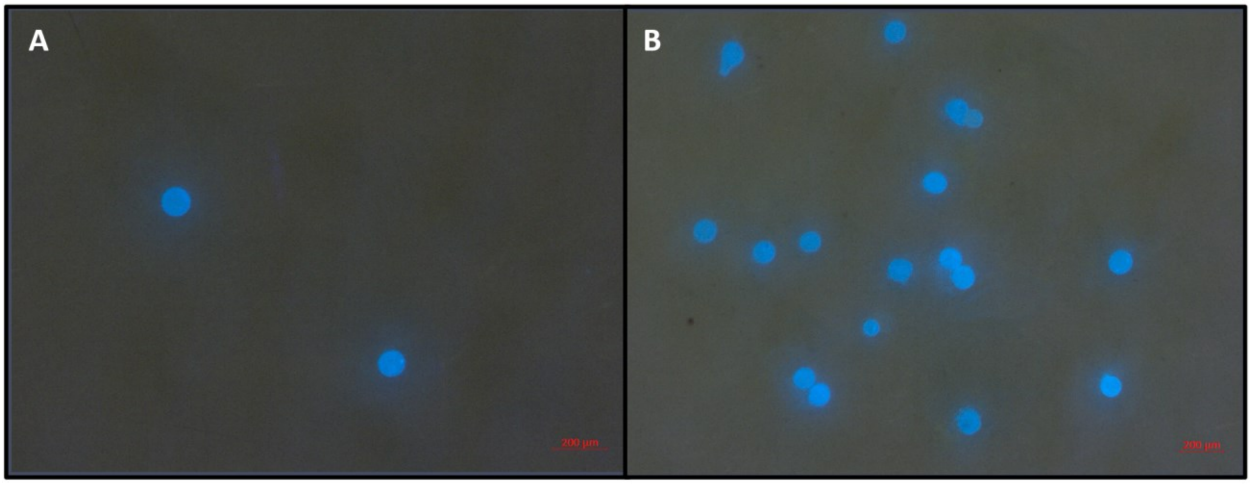

Figure S1: Blue fluorescent microplastic beads that were exposed to water and potassium hydroxide (KOH). Beads that were exposed to only water and underwent all filtering steps (A). Beads that were exposed to the 10% KOH for 2 days at 60°C and were filtered (B). There was no observable difference in the size or circularity of beads following the filtering or digestion process.

PROTOCOL

Protocol from Ritchie et al. Quantifying microplastic ingestion, degradation, and excretion in insects using fluorescent plastics. See manuscript for further details and recommendations.

Note to start: KOH is a hazardous chemical. Proper PPE guidelines should be used when handling KOH.

1. To start samples should be removed from the animal while keeping intact the digestive tract or tissue of interest. Tissues should be free of any water that may cling to them during dissection (to get an accurate weight). This can be done by gently touching them to a plastic-free cloth or wipe.
    a. If any tissues contain hard chitin or other components resistant to physical and/or mechanical breakdown, they can be manually broken open or the contents can be flushed into solution for downstream analysis.
2. Samples can be frozen for later digestion and filtration if needed.
3. Once samples are removed from the freezer, place them in a tube/container that can be used for KOH digestion (if not already). Ideally, this is a tube/container that is sealable as KOH will evaporate over time; if not sealable, place it in a fume hood while digestion is taking place.
    a. Note the amount of 10% KOH used should define before this step. Small tissue samples are better digested in a 1:10 ratio of KOH to mass, while larger tissue can be digested with a 1:3 ratio of KOH to mass.
4. While working in a fume hood that has been cleaned to limit possible plastic contamination, add the required amount of 10% KOH to the thawed samples in the digestion tube/container.
5. If taking place in a sealed container, KOH digestion can occur at 60°C and should be completed within approximately two days.
    a. Note that the temperature at which digestion takes place can be modified to suit the plastic polymer used, as some solvents are known to degrade some plastics at high temperatures.
    b. If not sealed, place tin foil over the container and leave it in a fume hood. It may take up to a week to allow for digestion to finish at room temperature.
6. After digestion, vortex each sample on at medium-high speed to allow for even distribution of plastic within digestion solution.
7. Working within a fume hood, pipette the digested solution (or a subsample of the homogenous mixture) and place it on a vacuum pump assembly equipped with the filter of choice.
8. Use the pump to remove all digestion solution from the sample, such that remaining particles are retained by the filter.
    a. Amount of time to remove solution will vary based on how much solution was placed on the vacuum assembly.

9. Once all fluid is removed, remove the filter, which should now contain plastics and minimal undigested organic material.
10. Carefully remove the filter from the assembly and place it in a container where airborne plastic particles cannot contact it, such as a petri dish with a lid.
    a. If the filter is correctly sealed from contamination, then photographing can be delayed, but if possible imaging should take place soon after sample filtration.
11. Place filters with isolated plastics under the view of a microscope, equipped for fluorescence microscopy (suitable for the excitation and emission spectra of the plastics used) and with a camera. Photograph sample on the filter.
    a. Important: A scale bar is required for proper measurements to take place in ImageJ
12. Loaded images into ImageJ and run through several automatic thresholds to capture the characteristics of plastics (refer to Ritchie et al for more details).
13. Measure the scale bar in ImageJ and set the image scale. This is done by placing the line cursor over the scale bar measurement
14. Split the image into RBG stacks to allow for noise removal
    a. This step depends on the fluorescence of plastic being used and the camera. Example, Blue fluorescence works best if the red stack is removed
15. Use the Z project function to recombine the remaining stacks of the image with it working by average intensity setting
16. Apply an auto threshold based on your sample's best fit.
    a. Intermodes and Renyientropy work well to capture large particles or small particles more accurately. An idea threshold can be determined by visual comparison to the original photo with a few samples.
17. Convert the photo to binary by the mask function after the auto threshold is applied. This changes the image for black particles on a white background.
18. Apply the watershed function to aid in the separation of close-together particles.
19. Use the analysis function to get the measurements of the plastic on the filter
    a. Adjust the infinity and the circularity to match what detection of particles best fit.
    b. If the shape is unknown, the circularity should be between 0-1.00.
20. If correctly followed this will generate a table and be saved as a CSV file to analyze the total amount of particles detected along with any parameters set such as Area.
